# Heritability of specific language impairment depends on diagnostic criteria

**DOI:** 10.1111/j.1601-183X.2007.00360.x

**Published:** 2008-04-01

**Authors:** D V M Bishop, M E Hayiou-Thomas

**Affiliations:** † Department of Experimental Psychology, University of Oxford Oxford, United Kingdom; ‡ Department of Psychology, University of York York, United Kingdom

**Keywords:** Etiology, heritability, specific language impairment, speech, twins

## Abstract

Heritability estimates for specific language impairment (SLI) have been inconsistent. Four twin studies reported heritability of 0.5 or more, but a recent report from the Twins Early Development Study found negligible genetic influence in 4-year-olds. We considered whether the method of ascertainment influenced results and found substantially higher heritability if SLI was defined in terms of referral to speech and language pathology services than if defined by language test scores. Further analysis showed that presence of speech difficulties played a major role in determining whether a child had contact with services. Childhood language disorders that are identified by population screening are likely to have a different phenotype and different etiology from clinically referred cases. Genetic studies are more likely to find high heritability if they focus on cases who have speech difficulties and who have been referred for intervention.

Specific language impairment (SLI) is a developmental disorder that is diagnosed when a child's language development is disproportionately poor relative to other skills for no apparent reason. Four twin studies have indicated that genes are important in the etiology of SLI (see Table 1). This can be inferred when monozygotic (MZ) twin pairs, who are genetically identical, are more concordant for disorder than are nonidentical, dizygotic (DZ) pairs, who share on average 50% of segregating alleles. In contrast, data from 4-year-old children from the Twins Early Development Study (TEDS) gave a different picture (Hayiou-Thomas *et al.*2005). Probandwise concordance for SLI was lower than in previous studies for both MZ and DZ twins, and heritability was negligible (see Table 1).

**Table 1:** Summary of studies that reported probandwise concordance rates for children with specific speech–language impairments

			Concordance	
			
Study	Sample (all same-sex twin pairs)	Age range (years)	MZ	DZ
Lewis & Thompson (1992)	32 MZ, 25 DZ pairs from Western Reserve Twin Project where at least one twin had had therapy. Most had articulation problems.	6–12	0.86	0.48
Bishop *et al.*(1995)	63 MZ and 27 DZ twin pairs; at least one twin met diagnostic criteria for specific speech or language impairment.	7+	0.70	0.46
Tomblin & Buckwalter (1998)	40 MZ and 22 DZ twin pairs plus three triplet sets, where at least one had low language test composite and normal IQ.	5–16	0.96	0.69
Hayiou-Thomas *et al.*2005	Subset of children from TEDS given in-home testing; 60 MZ and 55 DZ with one or both twins meeting criteria for SLI (language factor −1 SD or less and nonverbal ability better than −1 SD).	4	0.36	0.33
DeThorne *et al*. (2006)	248 twin pairs from Western Reserve Reading Project, including 165 children with parental report of problem in expressive (E) or receptive (R) language and/or articulation (A)	6	0.89	0.53 (E)
			0.67	0.20 (R)
			0.86	0.44 (A)

The current paper considers whether these discrepant findings might reflect variation in the ways in which samples were selected. With the exception of Hayiou-Thomas *et al*. (2005), the studies in Table 1 identified children on the basis that there had been clinical concern about one or both twins’ speech and language development. Lewis & Thompson (1992) used a parental questionnaire to identify twins who had received treatment of a speech–language problem, following up with a telephone interview to establish the nature of the problem. Bishop *et al*. (1995) and Tomblin & Buckwalter (1998) advertised for parents to volunteer their twin children if one or both had a speech–language problem. In the study by DeThorne *et al*. (2006) children were deemed affected if a parent reported that the child had had difficulties with speech or language. In contrast, Hayiou-Thomas *et al*. (2005) used a large-scale community sample, from which twin pairs likely to contain a child with language difficulties were identified through parental responses on a questionnaire about vocabulary size and language complexity, and the diagnosis of SLI was then made from scores on language and nonverbal tests administered at 4 years of age. The child's contact with speech and language pathology (SLP) services was not taken into account when identifying affected cases. This raises the possibility that discrepancies between findings from twin studies may be explained by a ‘clinical concern’ hypothesis, which maintains that heritability is high only in those children who arouse parental concern and/or are referred for SLP services. This hypothesis fits with an analysis of preschool parental questionnaire data from TEDS, where heritability of early language delay was higher when the phenotype was defined in terms of parental concern or professional contact than when parental report of vocabulary size or language complexity were used (Bishop *et al*. 2003).

Further data from TEDS were collected when the children were 7 years old, using parental questionnaire and telephone testing. We used these data to evaluate the clinical concern hypothesis, by comparing heritability for SLI when the impairment was defined on the basis of psychometric test results at 4 years, as compared with when contact with SLP services by 7 years of age was the basis for diagnosis.

## Methods

### Sample

TEDS is a longitudinal study of a community sample of twins born in England and Wales between 1994 and 1996. For a detailed account of the methods of sampling and assessment used in the initial phases of data collection, see Trouton *et al*. (2002). At 4 years of age, a subset of twin pairs was selected for individual language and cognitive assessment at home. This sample was selected to be overrepresentative of children at risk for language difficulties but also included twin pairs not deemed to be at risk. Overall, there were 191 MZ and 193 same-sex DZ pairs selected because one or both twins showed evidence of risk of language difficulties. This was determined on the basis of parental responses to a questionnaire completed when the child was 4 years of age. Language impairment (LI) risk was identified in children who were (1) not talking in full sentences; (2) had expressive vocabulary below the 15th centile or where (3) the parent was concerned because the child's language was developing slowly. A further 104 MZ and 103 DZ same-sex pairs were selected on the basis that neither twin showed signs of language difficulties (low-risk group). As in the Hayiou-Thomas *et al*. (2005) study, we excluded cases where the LI was associated with sensorineural hearing loss, physical handicap, autism or another syndrome affecting cognitive development and restricted ethnic status to white Caucasian with English as a first language (to minimize stratification effects in future molecular genetic studies of this sample). This gave a final sample of 333 pairs with an LI-risk child and 194 with low risk.

Parents of all children gave signed consent for participation. The study received approval from the Joint South London and Maudsley and the Institute of Psychiatry NHS Research Ethics Committee.

### Assessment

The in-home test battery given at 4 years of age is shown in Table 2. At 7 years of age, a measure of verbal ability from telephone testing (Harlaar *et al*. 2005) was available for 67% of the children who had been seen at 4 years. This consisted of the average score on the Vocabulary and Similarities subtests of the Wechsler Intelligence Scale for Children, 3rd edition (Wechsler 1997).

**Table 2:** Test battery given to children for in-home assessment at 4 years

Test	Authors
Language composite	
Bus story test, information	Renfrew (1988)
Action Picture Test, grammar	Renfrew (1988)
Verbal comprehension, British Ability Scales	Elliott *et al*. (1983)
Phonological awareness task (in-house eight-item test)	Viding *et al*. (2003)
Word knowledge, McCarthy Scales of Children's Abilities (MCSA)	McCarthy (1972)
Verbal fluency (MCSA)	McCarthy (1972)
Opposite analogies (MCSA)	McCarthy (1972)
Speech composite	
Goldman–Fristoe test of articulation	Goldman & Fristoe (1986)
Nonword repetition task (20-item version)	Gathercole *et al*. (1994)
Nonverbal composite	
Block Building (MCSA)	McCarthy (1972)
Puzzle Solving (MCSA)	McCarthy (1972)
Tapping Sequence (MCSA)	McCarthy (1972)
Draw-a-Design (MCSA)	McCarthy (1972)

### Classification of children

Children were first categorized into three groups on the basis of performance on the in-home test battery at 4 years of age, as described by Hayiou-Thomas *et al.*(2005), the only difference being that we included all children seen at 4 years, including low-risk as well as LI-risk pairs. A language composite was formed by averaging *z*-scores (computed relative to the low-risk pairs) on the first seven measures of language skills shown in Table 2. Note that two measures that require accurate speech production, Goldman–Fristoe articulation and nonword repetition were not included in this composite, as they had been found by Hayiou-Thomas *et al*. (2006) to load on a separate factor. A nonverbal composite was formed by averaging four nonverbal measures from the McCarthy Scales of Children's Abilities (McCarthy 1972) that had high loadings on a nonverbal factor: Block Building, Puzzle Solving, Tapping Sequence and Draw-a-Design (Viding *et al.*2003). Children who had *z*-scores less than −1 on both language and nonverbal composites were categorized as non-specific language impairment (NLI), and those who had language *z*-score less than −1, but nonverbal *z*-score better than −1 were categorized as SLI.

For an alternative analysis, children were categorized on the basis of parental report when they were 7 years of age according to whether they had ever had been referred to a speech–language pathologist for assessment or treatment.

## Results

### Overlap between different phenotype definitions

**Figure 1:**
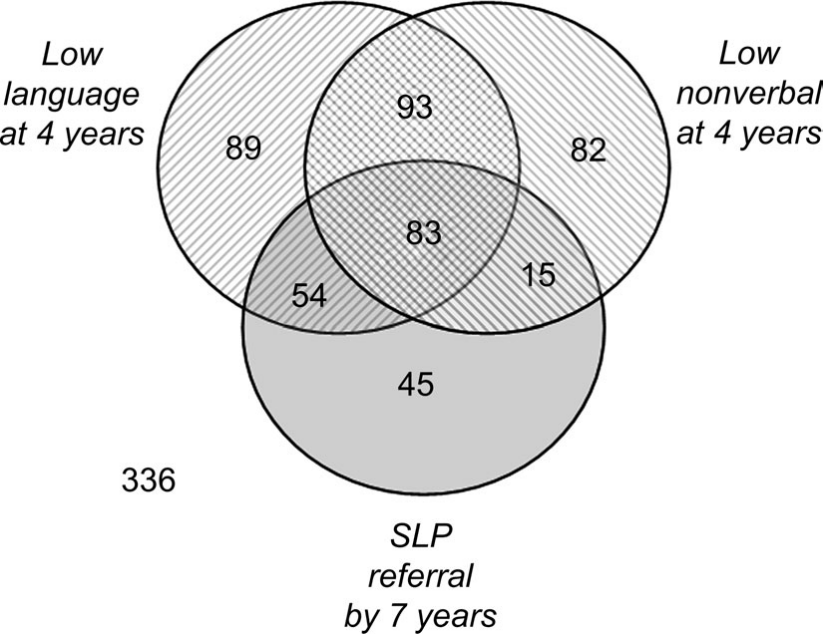
Classification of children at 4 years of age according to whether they had low language (score on composite from individual assessment of −1 SD or more below the mean), low nonverbal ability (analogous criterion for nonverbal scales) and whether they had contact with SLP services by 7 years of age Three hundred and thirty-six cases shown outside the set diagram did not meet criteria for any of these categories.

Figure 1 shows overlap between the categorization in terms of test scores at 4 years and in terms of SLP contact. Cases of SLI at 4 years are represented by the area of the set diagram, where low language does not intersect with low nonverbal. Only 54 (38%) of the 143 children meeting this criterion had been referred to SLP services by the age of 7 years. Figure 1 also shows children with nonverbal ability more than 1 SD (standard deviation) below the mean at 4 years of age: where these cases also fulfill criteria for low language, they correspond to the NLI category of Hayiou-Thomas *et al*. (2005). A higher proportion of these NLI cases, 83 of 176 (48%), had contact with SLP services, though this trend did not reach significance *χ*^2^ = 2.84, df = 1, *P* = 0.09. For children who did not meet criteria for low language, 45 of 381 (12%) of those with normal nonverbal ability and 15 of 97 (15%) of those with low nonverbal ability had contact with SLP services, a nonsignificant difference.

### Heritability estimates

Table 3 shows probandwise concordance rates for the children who were seen for in-home assessment at 4 years. Cases are categorized according to the original criterion used by Hayiou-Thomas *et al*. (2005) and according to contact with SLP services. There is a striking difference in results, with much higher concordance for MZ and DZ twins when impairment is categorized by SLP status, as compared to when it is diagnosed according to language test results. Heritability (*a*^2^) was estimated from tetrachoric correlations using mx software to fit a biometric model to the data including terms for additive genetic variance, shared environment and nonshared environment (Neale *et al*. 1999). This method is suitable when cases are classified into ordinal categories. It is potentially less sensitive than the DeFries–Fulker method that was used by Hayiou-Thomas *et al*. (2005) because it does not take into account the quantitative information on which the categories are based. Nevertheless, we used this method because only categorical information was available for classification of SLP status, and, in fact, the estimates obtained this way were very close to those reported by Hayiou-Thomas *et al*. Heritability estimates were high and statistically significant when SLP status was used to define the phenotype, but not when test data were used. Because the likelihood of the child having contact with SLP services was increased for those with low nonverbal ability (see Fig. 1), a further analysis was conducted excluding any pair where one or both twins had a nonverbal score more than 1 SD below the mean. This did not influence the heritability estimate (see Table 3).

**Table 3:** Probandwise concordance and heritability estimates with LI identified by test scores or by SLP contact

	Concordant probands	Total *n* probands	Probandwise concordance	Concordant probands	Total *n* probands	Probandwise concordance	*a*^2^	95% Confidence interval
				
Definition of LI	MZ			DZ			*a*^2^	95% Confidence interval
SLI on language tests	38	97	39%	24	79	30%	0.00	0–0.45
NLI on language tests	92	146	63%	40	95	42%	0.36	0–0.79
With SLP contact	94	108	87%	36	91	40%	0.96	0.70–0.99
With SLP contact, normal nonverbal	44	50	88%	16	46	35%	0.97	0.56–0.99

### Test profile for children who did and did not get referred for SLP

Having found that heritability was markedly higher for children who attracted clinical concern than for other children with LI, the next question was what was distinctive about those who were seen by SLPs. We considered two possible explanations. One possibility was that high heritability was a function of persistence of disorder. Thus, if transient problems resolve before professional help is sought and such problems are not heritable, this could explain the pattern of findings. Another possibility is that there is something distinctive about the phenotype of children who receive SLP services. There is evidence that overt problems with speech production are more likely than language difficulties to prompt clinical referral (Zhang & Tomblin 2000).

**Figure 2:**
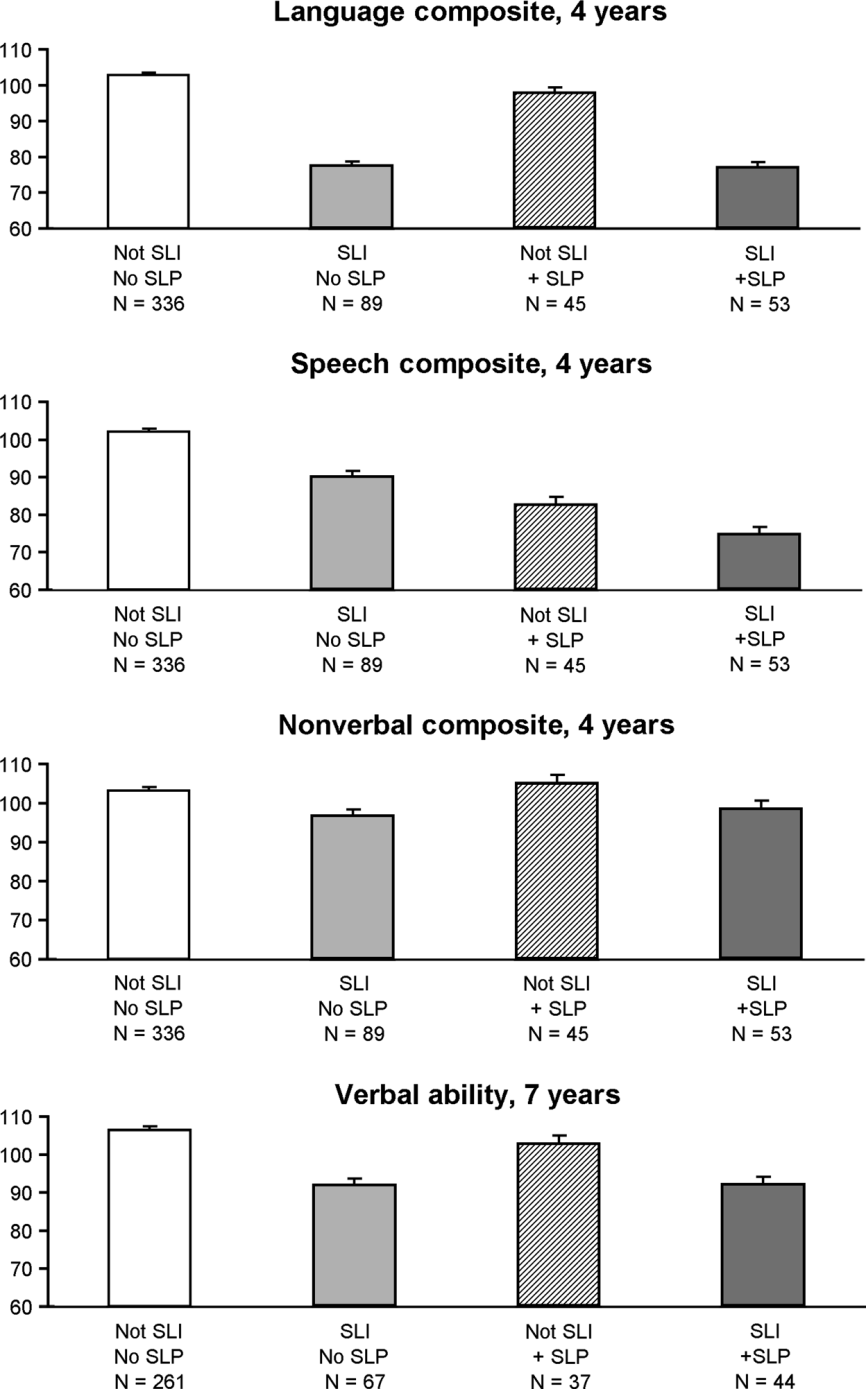
Mean scores on language, speech and nonverbal composites at 4 years and verbal ability at 7 years in relation to SLI status and referral to SLP Error bars show standard errors.

Children were subdivided using a two-way classification: whether or not they met the psychometric criteria for SLI used by Hayiou-Thomas *et al*. (2005) at 4 years of age and whether or not they had been referred to SLP by 7 years of age. Because our focus was on SLI, children with nonverbal ability more than 1 SD below average were excluded from this analysis. Mixed model analysis with family as a repeated measure was used to avoid problems arising from dependencies when two twins from a pair are included in the same analysis (Kenny *et al*. 2006); this adjusts the degrees of freedom in analysis of variance (anova) to account for statistical dependence between twins. Figure 2 shows mean scores on language, speech and nonverbal composite measures from assessments at 4 years, with data rescaled to mean 100 and SD 15. A series of anovas indicated that the factor SLI vs. no SLI had a significant effect on all three composites: language, *F*(1,460.3) = 411.4, *P* < 0.001, *η*^2^ = 0.472; speech, *F*(1,497.1) = 37.8, *P* < 0.001, *η*^2^ = 0.070; nonverbal, *F*(1,520) = 19.2, *P* < 0.001, *η*^2^ = 0.036. It is, of course, not surprising that there is a large effect on the language composite because this was used to define SLI.

Of greater interest is the effect of SLP referral. There was a significant effect of this factor on the language composite, *F*(1,516.2) = 4.5, *P* = 0.03, *η*^2^ = 0.008, and also a significant interaction between SLP referral and SLI status, *F*(1,459.6) = 3.86, *P* = 0.05, *η*^2^ = 0.008, but these were trivial in magnitude, as indicated by the effect sizes. The only measure for which this factor accounted for an appreciable amount of variance was the speech composite, where *F*(1,492.6) = 99.6, *P* < 0.001, *η*^2^ = 0.168. The interaction between SLI and SLP referral was not significant for this variable.

The verbal ability measure from telephone testing at 7 years of age was subjected to a parallel analysis. This measure correlated at *r* = 0.58 with the language composite at 4 years (*n* = 617, *P* < 0.001). Children who had been identified with SLI at 4 years did worse than other children on verbal ability: *F*(1,404.8) = 53.8, *P* < 0.001, *η*^2^ = 0.117. However, there was no difference on this measure between children who did and did not have referral for SLP and no interaction between SLI status and SLP referral.

Thus, the pattern of results showed that children who had SLP contact were comparable to other children in terms of severity of LI, both at 4 and 7 years, but those who had been referred did worse at 4 years on tests with a speech component.

### Heritability of speech impairment at 4 years

These results raised the question of whether higher heritability would be found at 4 years if the phenotype were defined in terms of impairment on speech measures rather than language measures. As noted above, two speech measures, Goldman–Fristoe articulation and nonword repetition, had been excluded from the definition of SLI by Hayiou-Thomas *et al*. (2005) because they loaded on a different latent factor from the other language measures. Hayiou-Thomas *et al*. (2006) reported a genetic correlation of 0.64 between these two factors. To consider genetic influences on impairment, we used DeFries–Fulker (1985) analysis to estimate group heritability 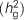 for a language composite and a speech composite derived from the 4-year-old test data. Again, children were excluded if either member of the twin pair had nonverbal ability more than 1 SD below the mean. Probands were defined in terms of having a score below cutoff on the relevant composite – language for the first three analyses and speech for the second three (see Table 4). The estimate of 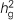 was significant for the speech composite only. Furthermore, 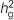 became larger as the cutoff for speech impairment became more stringent. It is also noteworthy that the number of probands at the extreme cutoff of −2 SD was twice as great for the speech composite as for the language composite. This reflected the fact that, despite transformation of the data, the speech composite had significant positive skew [skew = −0.525, standard error (SE) = 0.152].

**Table 4:** Heritability estimates from DeFries–Fulker analysis on 4-year-old language and speech composites

	*n* probands		Cotwin mean*				
					
	MZ	DZ	MZ	DZ	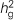	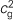	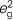
Language composite							
−1 SD cutoff	60	54	0.69	0.52	0.34 (0–0.78)	0.35 (0–0.68)	0.31 (0.15–0.45)
−1.5 SD cutoff	26	30	0.67	0.61	0.11 (0–0.63)	0.55 (0.14–0.77)	0.33 (0.13–0.49)
−2 SD cutoff	9	12	0.53	0.52	0.01 (0–0.76)	0.51 (0–0.71)	0.47 (0.18–0.66)
Speech composite							
−1 SD cutoff	67	59	0.76	0.57	0.38 (0.01–0.75)	0.37 (0.07–0.67)	0.24 (0.11–0.37)
−1.5 SD cutoff	43	37	0.79	0.48	0.62 (0.21–0.93)	0.17 (0–0.49)	0.21 (0.07–0.35)
−2 SD cutoff	24	16	0.87	0.32	0.84 (0.52–0.98)	0 (0–0.25)	0.16 (0.02–0.31)

* Scores transformed so that proband means = 1.

## Discussion

We started with the observation that results from Hayiou-Thomas *et al*. (2005) were inconsistent with previous studies, in that low and nonsignificant estimates of heritability for SLI were obtained in a sample of 4-year-olds. Much higher heritability was found in these same 4-year-olds when SLI was redefined in terms of referral to SLP services, ruling out an explanation for the low heritability in terms of the young age of twins. This suggested that there is something distinctive about the phenotype in children who attract clinical concern.

One possibility was that children with SLP referral simply had more severe or persistent language problems than other cases. It could be argued that many of those meeting criteria only on language tests had mild or transient problems, or may indeed turn out to be ‘false positives’, whose low scores reflected error of measurement and would regress to the mean on retest (cf. Zhang & Tomblin 2003). Data from a later wave of assessment confirmed that regression occurred, with mean language scores improving over time. However, there was no evidence that children with SLP referral had more severe or more persistent language problems than other cases. Rather, they appeared to have a qualitatively different profile of impairment, with poor performance at 4 years of age on tests where accuracy of speech production was crucial.

This finding led us to return to the 4-year-old data and carry out genetic analysis of extreme scores for separate speech and language composites. Only the former was significantly heritable, with group heritability estimates increasing as the cutoff for disorder was made more stringent. This pattern of results, coupled with the skewed distribution of scores on the speech composite, is what is expected if speech impairment is caused by a single gene of major effect (Bishop 2005), although other mechanisms, such as gene–environment interaction, could also provide an explanation. The opposite pattern was seen for the language composite, with heritability declining as the cutoff became more extreme; however, it must be noted that the estimates of heritability are based on tiny numbers, and the standard errors are correspondingly large, so this result must be interpreted with caution.

At first glance, the negligible heritability seen for low scores on the language composite appears to contradict other analyses of the same data set reported by Kovas *et al*. (2005). They reported genetic analyses of the individual language tests obtained from 4-year-olds and showed modest but significant heritability for most measures, both on standard individual differences analysis and on liability-threshold analysis (which considers heritability of extreme scores). Note, however, that the analyses conducted by Kovas *et al.* did not take nonverbal ability into account. There are strong correlations between verbal and nonverbal ability, and it is clear that many genetic influences are non-specific, affecting both verbal and nonverbal measures (Kovas & Plomin 2006). If children with low nonverbal ability are included in extremes analysis, estimates of heritability tend to be higher, presumably because they incorporate the effects of generalist genes as well as those exerting more specific effects. Furthermore, the cutoff for extremes used by Kovas *et al.* was set at −1 SD; in our analyses, the clearest differences between language and speech measures were seen at a more extreme cutoff than this (see Table 4).

The mismatch that we found between recognition of SLI by clinicians and diagnosis based on psychometric tests is in line with other studies. In an epidemiological survey, Tomblin *et al*. (1997) found that only 29% of children who met criteria for SLI on a psychometric test battery had previously been identified as language impaired. Conversely, between 20% and 50% of children who were considered to have SLI based on clinical diagnosis had impaired performance on standardized language assessments (Aram *et al*. 1993; Ziegler *et al*. 1990). A study carried out in the Netherlands found that most preschool children receiving SLP had average or above-average scores on standardized language tests (Goorhuis-Brouwer & Knijff 2003). Such findings might suggest that clinical impression of speech impairment and LI is less accurate than formal testing. However, our data show that clinical impression is better than psychometric tests at identifying those children who have a heritable disorder. This suggests that there are aspects of a child's speech impairment and LI that cause concern to parents and professionals and are not adequately captured by psychometric tests (Dunn *et al*. 1996), yet are of etiological significance. To date, studies investigating this mismatch have focused more on features of language than of speech, with research suggesting that children who attract clinical concern are distinguished by impairments on measures from naturalistic speech samples, such as mean utterance length or proportion of structural errors (Dunn *et al*. 1996) or ratings of pragmatic abnormalities (Bishop 1998; Conti-Ramsden *et al*. 1997). The current study, however, emphasizes speech difficulties as a key feature that distinguishes children who are referred for SLP.

Zhang & Tomblin (2000) found that the presence of difficulties affecting speech production had a strong influence on whether or not a child was referred. We confirmed this pattern in our data and also showed that the presence of speech problems rather than LI is a phenotypic signature of a heritable disorder. This is consistent with previous work pointing to an important role for genetic factors in speech sound disorders (SSD). Several studies have demonstrated familial aggregation of SSD, such that the parents and siblings of probands are much more likely to also have some from of speech, language or literacy disorder than the population base rates (Campbell *et al*. 2003; Lewis & Freebairn 1998; Lewis *et al*. 2004). Twin studies that have examined speech and language difficulties separately have consistently found high heritability for speech problems. Lewis & Thompson (1992) included a large number of children with speech difficulties in their sample; the concordance rates for this subgroup of children were nearly perfect for MZ pairs (95%) and very low for DZ pairs (22%). Bishop *et al*. (1995) examined concordance rates for subtypes of speech and language disorder and found that the greatest MZ–DZ differences were for children with articulation disorder and expressive language deficits, with no evidence of genetic influence on pure receptive language disorder. Similar results were reported by DeThorne *et al*. (2006) (see Table 1).

Other work using quantitative measures has pointed to high heritability for percentage consonants correct, a measure of articulation (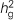 = 0.97; Bishop 2002), and for speech problems rated by parents and teachers (*h*^2^ = 0.91; Bishop *et al*. 2006b). Nonword repetition, which is designed not only to tap phonological memory processes but also makes substantial demands in terms of speech output, has consistently been found to be highly heritable in several different twin samples (Bishop 2002; Bishop *et al*. 1999, 2006a).

The results from these twin studies are also supported by an adoption study that compared probands to their biological family and to their adoptive (environmental) family. Having an affected biological parent was the best predictor of a child's speech-impaired status (Felsenfeld & Plomin 1997).

Finally, we may note that molecular genetic studies of both SLI and reading disability have confirmed that phenotypes based on speech production and/or nonword repetition appear especially likely to reveal significant linkage (SLI Consortium 2004; Smith *et al*. 2005; Stein *et al*. 2004).

Overall, these findings challenge the widely held view that a battery of language tests is necessarily the best way to measure a heritable phenotype. Language tests have the advantage of being objective and having known psychometric properties. All else being equal, composite scores based on factor analysis should be particularly useful as they will be more robust and have lower error variance than individual tests. However, our study indicates that they can miss key features of the heritable phenotype. We would not want to claim that there are no genes that have specific influences on aspects of language development; indeed Bishop *et al*. (2006a) found strong genetic influence on a measure of use of verb inflections. Although children with low nonverbal IQ were not excluded from that sample, the results could not be explained in terms of generalist genes because nonverbal IQ for those with poor verb morphology (mean = 97.7, SD = 10.5) was comparable to the rest of the sample. Our argument, rather, is that for many language measures, genetic influence may be part of more generalist influences on cognition and that one will therefore not find high heritability of LI once those with low IQ are excluded. Our data further suggest that the likelihood of identifying alleles implicated in disorder will be increased if we define the phenotype in terms of speech impairment. Many of the children selected by this method will also have comorbid LI. However, generalized LIs in children of normal nonverbal ability with normal speech appear largely environmental in origin.
